# Host–Pathogen Interactions in Cystic Fibrosis Lung Disease: Adaptation, Persistence, and Clinical Implications of *Pseudomonas aeruginosa*

**DOI:** 10.3390/pathogens15050557

**Published:** 2026-05-21

**Authors:** Burcu Capraz Yavuz

**Affiliations:** 1 Pediatric Pulmonology Department, Ankara Bilkent City Hospital, 06800 Ankara, Türkiye; caprazburcu@gmail.com; Tel.: +90-5336800633; 2 Department of Translational Medicine, Institute of Health Sciences, Ankara Yildirim Beyazit University, 06800 Ankara, Türkiye

**Keywords:** cystic fibrosis, *Pseudomonas aeruginosa*, host–pathogen interactions, chronic infection, bacteriophage therapy

## Abstract

Cystic fibrosis (CF) lung disease is characterized by chronic infection and progressive airway damage, driven by interactions between epithelial dysfunction, immune dysregulation, and microbial adaptation. Defective *cystic fibrosis transmembrane conductance regulator* (CFTR) function disrupts airway hydration and mucociliary clearance, creating a microenvironment that facilitates infection, particularly with *Pseudomonas aeruginosa* (*P. aeruginosa*). Within this environment, *P. aeruginosa* undergoes adaptive changes, including biofilm formation and metabolic reprogramming, which support long-term survival in the airway. Concurrently, host immune responses become dysregulated, with ineffective bacterial clearance and sustained neutrophil-dominated inflammation contributing to tissue injury. These processes establish a self-reinforcing cycle that drives disease progression. Importantly, early infection represents a critical therapeutic window during which bacterial populations remain more amenable to eradication before irreversible airway remodeling occurs. Delayed intervention promotes transition to a more treatment-refractory state and accelerates disease progression. Despite the clinical benefits of CFTR modulators, airway damage and established infections often remain. The relative contributions and interactions of epithelial dysfunction, immune dysregulation, and bacterial adaptation in sustaining chronic infection remain incompletely defined, representing a key knowledge gap. In this context, this review aims to integrate current evidence on host–pathogen co-adaptation in CF lung disease, with a particular focus on *P. aeruginosa*, and highlight emerging therapeutic strategies.

## 1. Introduction

Cystic fibrosis (CF) is a life-limiting autosomal recessive disorder caused by pathogenic variants in the *cystic fibrosis transmembrane conductance regulator* (*CFTR*) gene. Globally, approximately 160,000 individuals are estimated to be living with CF [1]. Pulmonary complications represent a major determinant of morbidity and mortality [2].

The hallmark of CF lung disease is a complex and self-perpetuating cycle of impaired mucociliary clearance (MCC), chronic infection, and inflammation [3,4]. Concomitantly, disruption of the airway epithelial barrier contributes to an aberrant host immune response to infection [5].

*Pseudomonas aeruginosa* (*P. aeruginosa*) is one of the most important airway pathogens when it comes to disease progression. Chronic infection with *P. aeruginosa* is associated with an increased frequency of pulmonary exacerbations, accelerated decline in lung function, and reduced survival [6,7]. Its presence has been linked to a twofold increase in mortality risk in people with CF (pwCF), rising to more than eightfold in the context of antibiotic-resistant strains [8,9].

Data from the European registry indicate that nearly 60% of patients with chronic *P. aeruginosa* infection prior to elexacaftor/tezacaftor/ivacaftor (ETI) remain infected after 1 year [10,11]. Multidrug-resistant *P. aeruginosa* also remains clinically relevant with a reported prevalence of 3.2% in 2024. Reduced frequency of airway culture sampling may lead to underestimation of the true prevalence of chronic *P. aeruginosa* infection [12,13].

Bacterial persistence in the CF airways may result either from the incomplete impact of these therapies on underlying mechanisms of disease or from the pre-existing structural airway damage [11,14,15,16]. Moreover, a substantial burden of chronic infection persists among pwCF who are ineligible for, unable to access, or unable to use these therapies [17]. These limitations emphasize the need for a deeper understanding of host–pathogen interactions. Such insight is essential to explain the persistence and complexity of *P. aeruginosa* infection in pwCF. In this context, this review aims to integrate current evidence on host–pathogen co-adaptation in CF lung disease, with a particular focus on *P. aeruginosa*, and highlight emerging therapeutic strategies.

This narrative review was conducted through a structured literature search of electronic databases, including PubMed, Scopus, and Web of Science. Articles published in English up to March 2026 were considered. Search terms included combinations of ‘cystic fibrosis,’ ‘*Pseudomonas aeruginosa*,’ ‘CFTR dysfunction,’ ‘airway microenvironment,’ ‘host–pathogen interactions,’ ‘biofilm,’ ‘innate immunity,’ ‘microbiome,’ ‘antimicrobial resistance’ and ‘inflammation.’ Original research articles, clinical studies, and relevant reviews were included based on their relevance to CF lung disease, microbial adaptation, and therapeutic strategies. Studies focusing on nonrespiratory manifestations of CF or lacking clear methodologic quality were excluded. Additionally, references were identified through manual screening of reference lists.

## 2. The Cystic Fibrosis Airway: A Unique Ecological Niche

### 2.1. Ion Channel Dysfunction and the Airway Microenvironment in Cystic Fibrosis

The airway microenvironment in CF is profoundly shaped by defects in epithelial ion transport, which are central to the pathogenesis of CF lung disease [18]. In this context, integrating experimental assays with clinical measures supports a comprehensive model in which CFTR loss initiates mucus hyperconcentration, further enhanced by mucin interactions and hypoxic inflammatory feedback [19].

Under physiologic conditions, airway hydration is maintained by a tightly regulated balance between CFTR-mediated chloride (Cl^−^) secretion and epithelial sodium channel (ENaC)-dependent sodium (Na^+^) absorption. In CF, loss of CFTR function reduces Cl^−^ secretion and leads to ENaC hyperactivity, resulting in excessive Na^+^ absorption and osmotic depletion of airway surface liquid (ASL) [20,21,22]. Beyond its classic role in ion transport, CFTR dysfunction should be considered within a broader biologic context. Recent systems biology and transcriptomic analyses suggest that CFTR functions within a complex regulatory network, where its disruption propagates across multiple signaling pathways [22,23]. For instance, altered calcium homeostasis, mitochondrial dysfunction, and impaired autophagy collectively contribute to defective epithelial stress responses and may promote microbial persistence [24,25].

Host–pathogen interactions further exacerbate these abnormalities. *P. aeruginosa* can directly interfere with CFTR regulatory mechanisms, including destabilization of scaffold proteins such as NHERF1, thereby reducing CFTR surface expression and amplifying epithelial dysfunction [26,27,28]. This bidirectional interaction highlights the dynamic nature of host–pathogen co-adaptation in the CF airway. However, the precise molecular mechanisms underlying these interactions remain incompletely understood and warrant further investigation.

Such a network-based perspective may help explain phenotypic variability among patients and contribute to personalized therapeutic strategies, taking into account that environmental and genetic modifiers can also play a significant role [23,25,29].

### 2.2. Immune Dysregulation in Cystic Fibrosis

Recent evidence indicates that in pwCF, epithelial integrity is compromised and the airways exhibit a primed state, predisposing the lung to exaggerated inflammatory responses even before infection is established [30,31].

Neutrophil-derived products, including elastase, reactive oxygen species (ROS), extracellular DNA, and actin, contribute to increased mucus viscosity and structural lung damage. Free neutrophil elastase levels strongly correlate with lung function decline, underscoring its clinical relevance. In addition, IL-17-producing neutrophils have been associated with worse pulmonary outcomes, suggesting that neutrophilic phenotypic heterogeneity may further influence disease progression [32,33,34,35]. Likewise, neutrophil extracellular traps (NETs), although antimicrobial in acute settings, predominantly exacerbate tissue injury in chronic CF airways [36,37]. Neutrophils release calprotectin, a cytosolic protein that accounts for 40–60% of their protein content, which limits bacterial dissemination by chelating essential divalent cations such as zinc and manganese. However, this localized cation depletion, together with the presence of anionic extracellular DNA, alters the mucus environment. These changes activate bacterial regulatory systems, such as PhoP-PhoQ, which induces lipid A modifications and enhances resistance to cationic antimicrobial peptides [38,39].

Beyond neutrophils, epithelial-derived cytokines such as interleukin (IL)-1β and IL-1α play a central role in amplifying mucus hypersecretion. These cytokines activate downstream pathways, including SPDEF and ERN2, linking inflammatory signaling to mucus production [40]. Consequently, immune activation becomes directly linked to structural airway changes, thereby accelerating disease progression.

Moreover, macrophages in CF exhibit profound functional abnormalities, including impaired phagocytosis, defective efferocytosis, and a sustained proinflammatory phenotype [41]. This dysregulation is further compounded by altered polarization dynamics, with a shift toward M1-like responses [42,43]. Importantly, these defects appear to arise from both intrinsic CFTR dysfunction and the chronically inflamed airway microenvironment, highlighting the bidirectional nature of host–pathogen interaction. The therapeutic efficacy of future treatments such as phage therapy may also be constrained by these immune cells. Alveolar macrophages have been shown to phagocytose bacteriophages, thereby reducing their local density and bioavailability in the lung. This finding highlights the need for optimized delivery routes that minimize immune-mediated clearance [44].

In parallel with host immune dysregulation, recent evidence suggests that *P. aeruginosa* evades host immunity and actively subverts it through the secretion of the metalloprotease LasB. This toxin cleaves the host-derived growth factor amphiregulin into a bioactive form that induces type 2-related genes and the mucin Muc5ac. By diverting the host response from a protective antibacterial (Type 3) profile toward a suboptimal Type 2 response, the pathogen promotes excessive mucus production. Crucially, this mucus serves as a primary nutrient source, allowing the bacterium to create and sustain its own supportive ecologic niche within the lung [45,46].

At the cellular level, CFTR dysfunction also impairs defense mechanisms. Reduced CFTR expression disrupts NRF2-mediated antioxidant responses and alters redox balance, and ceramide accumulation interferes with lysosomal function and autophagy [47]. These intracellular alterations further sustain cellular stress [48,49].

Furthermore, pathogen-associated molecular patterns (PAMPs) and damage-associated molecular patterns (DAMPs) drive inflammasome activation and NF-κB signaling, resulting in excessive production of proinflammatory cytokines and amplification of oxidative imbalance [50].

Adaptive immune responses are also affected, with a skewing toward Th17-driven inflammation and a relative deficiency of regulatory T cells (Treg). This imbalance contributes to inadequate immune regulation [51]. Both CFTR dysfunction and *P. aeruginosa* infection have been associated with reduced Treg numbers, which correlate with disease severity [52]. This adaptive immune imbalance is closely linked to preceding innate immune activation. Persistent antigen exposure and dendritic cell dysfunction impair effective T cell priming and promote polarization toward pro-inflammatory phenotypes. Th17-driven responses further amplify neutrophilic recruitment and epithelial dysfunction [36]. In addition, emerging evidence suggests that B cell responses are quantitatively increased but functionally insufficient, contributing to ongoing antigen stimulation without effective bacterial eradication [36].

Collectively, innate and adaptive immune abnormalities in CF create a self-perpetuating inflammatory environment that promotes microbial persistence and progressive lung damage. These pathways are closely linked to CFTR dysfunction, but they also involve complex secondary mechanisms. Therefore, effective therapeutic strategies may require combined approaches targeting both epithelial defects and immune dysregulation.

## 3. Early vs. Chronic Infection: The Turning Point

**Figure 1:**
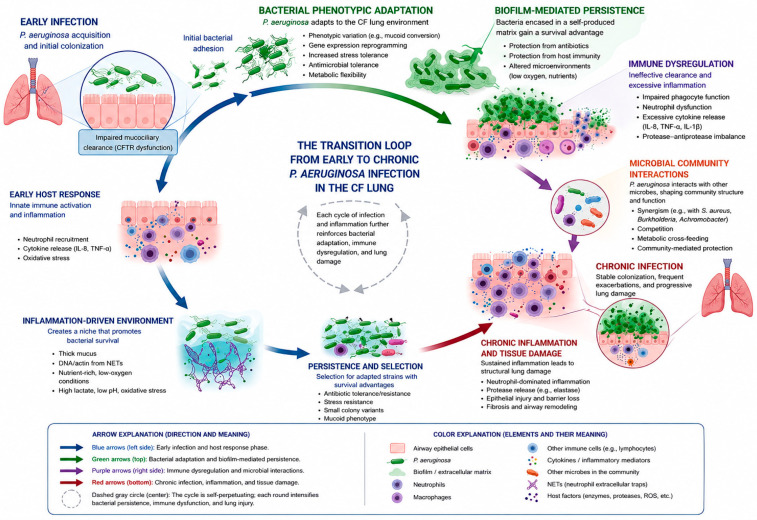
Transition from early *P. aeruginosa* to chronic infection in cystic fibrosis airways.

The transition from early *P. aeruginosa* acquisition to chronic infection represents a critical pathobiologic inflection point in CF lung disease. This transition is not simply defined by increasing bacterial burden; rather, it reflects a dynamic ecologic and evolutionary remodeling of the airway microenvironment (Figure 1).

As conceptualized in Figure 1, early infection is characterized by planktonic, metabolically active bacterial populations. These organisms retain acute virulence traits and remain susceptible to both antibiotic therapy and host immune clearance [53]. In contrast, chronic infection represents a fundamentally distinct state, metabolic reprogramming, immune evasion, and progressive structural airway damage [54].

However, Douglas et al. reported an 18.2% prevalence of mucoid *P. aeruginosa* at first isolation in screened young children, suggesting that early mucoid forms might occur without prior phenotypic transition [55]. Together, these findings indicate that progression to chronic infection is heterogeneous, not linear, and may occur earlier than traditionally assumed. However, the precise determinants and timing of this inflection point remain incompletely understood and likely vary across pwCF.

### 3.1. Initial Acquisition of Pseudomonas aeruginosa

Initial acquisition of *P. aeruginosa* in CF can occur from environmental reservoirs as well as through patient-to-patient transmission [56]. Early isolates exhibit a phenotype optimized for colonization and invasion, retaining functional motility structures such as flagella and type IV pili. They also possess an intact type III secretion system (T3SS) capable of delivering effector proteins, including ExoS or ExoU, into host cells [57,58].

These virulence factors promote epithelial disruption, cytotoxicity, and immune activation of innate responses. At this stage, bacterial populations are metabolically active with quorum sensing systems, coordinating the expression of key virulence determinants such as elastase and pyocyanin [59].

**Figure 2:**
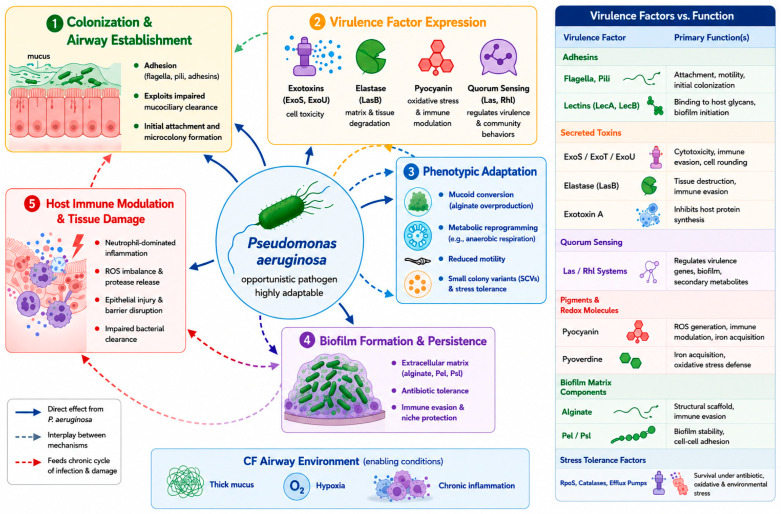
Virulence factors and host–pathogen co-adaptation of *P. aeruginosa* in the cystic fibrosis airway.

In non-CF airways, such acute virulence traits would typically result in effective microbial clearance. However, in CF, as discussed previously, impaired MCC, airway surface dehydration, reduced antimicrobial activity, and defective phagolysosomal killing create a permissive airway microenvironment that enables bacterial persistence. Figure 2 integrates these host-related abnormalities, together with microbial virulence mechanisms, highlighting the early stages of host–pathogen interaction.

Importantly, early infection represents a critical therapeutic window during which eradication strategies are most effective. Once this transition is established, eradication potential declines substantially, and infection becomes increasingly refractory to both immune clearance and antimicrobial therapy [60,61].

### 3.2. Adaptation and Phenotypic Switching

The adaptation of *P. aeruginosa* within the CF airway reflects the cumulative impact of persistent and overlapping selective pressures that reshape bacterial phenotype and function [62]. These pressures, including hypoxia within mucus plugs, oxidative stress, repeated exposure to antibiotics, and nutrient heterogeneity, collectively drive a shift toward persistence rather than acute virulence [63,64].

In response to these conditions, *P. aeruginosa* undergoes coordinated phenotypic adaptation characterized by reduced expression of acute virulence determinants. Flagellar expression is reduced, thereby decreasing immune recognition via toll-like receptor 5. T3SS activity is also downregulated [65]. The transition from acute to chronic infection is governed by a complex hierarchy of two-component systems that sense environmental cues. In this context, the GacS-GacA system serves as a master switch. When activated by signals such as calcium or inhibited by mucin glycans via RetS, it regulates the production of small RNAs (RsmY and RsmZ) that sequester RsmA/N proteins. High levels of these small RNAs favor a chronic phenotype characterized by type VI secretion system activity. At the same time, acute virulence factors such as the T3SS and motility are downregulated. Additionally, the oxygen-responsive small RNA SicX has been identified as a critical “chronic-to-acute” switch during mammalian infection. This mechanism allows the bacteria to adapt to fluctuating oxygen levels within mucus plugs. Importantly, these phenotypic changes are underpinned by genetic diversification and regulatory reprogramming. Mutations in quorum sensing regulators, particularly *lasR*, further reprogram virulence expression and metabolic pathways. Concurrently, activation of the AlgU regulon promotes alginate overproduction and the emergence of the mucoid phenotype, a hallmark of chronic CF infection [66,67].

Notably, these adaptive processes occur within a highly dynamic and heterogeneous bacterial population. Multiple genotypically distinct *P. aeruginosa* lineages can coexist within the same patient, even within a single sputum sample. This finding indicates ongoing diversification rather than clonal uniformity and suggests parallel evolutionary trajectories during chronic infection [68].

## 4. Biofilm Formation and Biofilm-Mediated Persistence

Biofilm formation is a defining feature of chronic *P. aeruginosa* infection in CF and a key driver of long-term bacterial survival. Within the CF airway, bacteria form structured, matrix-embedded communities composed of alginate, Pel, and Psl polysaccharides, proteins, and extracellular DNA derived from both bacterial and host neutrophil sources [69]. This organization reshapes bacterial physiology and alters host–pathogen interactions [69,70].

Biofilm-mediated persistence in the CF airway is increasingly understood as a form of phenotypic tolerance driven by the extracellular polymeric substance matrix. Limited antibiotic penetration further contributes to reduced treatment efficacy [71]. Biofilm-associated tolerance mechanisms, including extracellular matrix-mediated diffusion barriers, efflux pump activation, and metabolically dormant subpopulations, play a central role [72,73].

For instance, extracellular DNA serves as a potent chelator of divalent cations, thereby activating the PhoPQ and PmrAB regulatory systems. This process leads to modified lipopolysaccharide expression that effectively blocks aminoglycoside uptake [74]. Furthermore, the M-rich alginate characteristic of mucoid strains can physically sequester cationic antibiotics such as tobramycin. This ‘shielding effect’ creates distinct regions within the biofilm where deep-seated bacterial populations remain untouched by therapy, despite showing susceptibility in standard in vitro planktonic cultures [74].

Regulatory networks also play a critical role in maintaining these structural and metabolic adaptations. Quorum sensing and c-di-GMP signaling dynamically coordinate biofilm formation and maintenance, promoting a shift from acute virulence to chronic persistence. Elevated c-di-GMP levels drive the transition toward a sessile phenotype and increased matrix production [75].

Furthermore, intra-host diversification collectively improves adaptive capacity in the face of antibiotic and immune pressure. It is increasingly acknowledged as a crucial factor in disease progression, although the specific impact of individual subpopulations on clinical outcomes is not yet fully elucidated [76].

## 5. The Hyperinflammatory Loop and Immune Dysregulation in Cystic Fibrosis

*P. aeruginosa* infection drives a self-amplifying inflammatory loop in CF, in which host defense mechanisms fail to eradicate infection and promote airway damage. This process reflects a state of immune dysregulation, where antimicrobial responses become maladaptive and contribute to disease progression.

Bacterial components, including lipopolysaccharide, flagellin, and pyocyanin, activate epithelial NF-κB signaling and sustain IL-8 production, leading to continuous neutrophil recruitment. However, neutrophil influx does not result in effective bacterial clearance [77]. Neutrophil elastase further contributes to tissue damage by degrading extracellular matrix proteins, disrupting epithelial junctions. It also impairs ciliary function, and cleaves immune receptors, thereby reducing bacterial killing capacity [78,79,80]. Epithelial barrier disruption increases paracellular permeability and facilitates deeper persistence within the airway [81,82,83,84].

As a result, the innate immune response remains persistently activated but functionally ineffective [85,86]. Clinically, this hyperinflammatory loop and immune dysregulation are associated with recurrent pulmonary exacerbations and progressive decline in lung function [84]. In this context, recent studies highlight the potential value of combined therapeutic strategies targeting both immune dysregulation and microbial persistence [54,87,88,89].

## 6. Microbial Evolution and Community Interactions in the CF Airway

The CF airway constitutes a highly dynamic eco-evolutionary system in which genetic adaptation and community-level interactions occur concurrently. Rather than representing independent processes, within-host evolution and polymicrobial ecology are interdependent and collectively influence disease trajectory [83,90].

Longitudinal analyses have demonstrated that chronic *P. aeruginosa* populations acquire mutations in regulatory networks controlling signaling pathways, exopolysaccharide production, antimicrobial susceptibility, and secretion systems. These genetic changes promote a transition toward phenotypes optimized for long-term residence within the airway environment. Notably, emerging evidence indicates that such evolutionary trajectories are shaped both by host-derived pressures and interactions with coexisting microorganisms [91,92,93].

CF airway harbors a complex microbial ecosystem, particularly in early disease stages. Diverse bacterial taxa, along with viral and fungal components, contribute to a structured community that evolves over time. As disease progresses, community composition shifts toward reduced diversity and increased dominance of highly adapted organisms [94]. This ecologic transition is associated with worsening clinical status, although causality remains difficult to establish [95]. Evidence from a comprehensive 21-year analysis demonstrates that chronic *Staphylococcus aureus* (*S. aureus*) and *P. aeruginosa* coinfection is associated with better respiratory function tests and fewer intravenous antibiotic days than chronic *P. aeruginosa* monoinfection. This may occur because *S. aureus* suppresses *P. aeruginosa*’s virulence or only less aggressive *P. aeruginosa* strains tolerate coinfection, highlighting that these pathogens influence each other’s impact on the host [96]. Consequently, understanding these complex microbial interactions and the chronicity of infection is essential for developing more precise and effective treatment strategies for pwCF.

Social dynamics within these microbial populations also influence stability and virulence. Quorum sensing deficient subpopulations, such as *lasR* mutants, may exploit shared extracellular products without contributing to their production, thereby reducing the metabolic burden associated with cooperative behaviors. Although expansion of these variants has the potential to destabilize population structure, their persistence is frequently limited by regulatory mechanisms that maintain cooperative balance within the microbial community [67,97].

In addition to bacterial interactions, respiratory viruses can modify epithelial susceptibility and microbial adherence, and fungal species influence nutrient availability and immune signaling. However, the extent to which these interactions directly modulate long-term disease progression remains incompletely defined [98].

Polymicrobial interactions significantly complicate diagnostic accuracy, particularly regarding co-infections with *Aspergillus fumigatus*. The presence of *P. aeruginosa* has been found to reduce fungal culture positivity by nearly 40%, primarily through siderophore-mediated competition (e.g., pyoverdine) and phenazine-induced growth inhibition. This creates an ‘invisible’ fungal burden in samples that are sequencing-positive but culture-negative (S+/C−), which may lead to the underestimation of fungal prevalence and subsequent clinical mismanagement [99].

Furthermore, the functional relevance of anaerobes (e.g., *Prevotella*, *Veillonella*) in the CF lung extends to mechanisms of passive resistance. Many of these organisms produce extracellular β-lactamases that degrade antibiotics in the local environment, thereby indirectly protecting otherwise sensitive *P. aeruginosa* from eradication. Additionally, the fermentation of host mucins by anaerobes generates short-chain fatty acids, such as acetate and butyrate. These metabolites can stimulate neutrophil chemotaxis and IL-8 production, potentially fueling the chronic hyperinflammatory loop even in the absence of acute aerobic blooms [100].

## 7. Impact of CFTR Modulator Therapy on Host–Pathogen Interactions

CFTR modulator therapy (CFTRm) has transformed the clinical landscape of CF by restoring epithelial ion transport. These agents improve airway surface hydration, enhance MCC, and normalize airway pH. As a result, several conditions that favor microbial establishment are mitigated. Despite the transformative impact of CFTRm, clinical data indicate that established infections are difficult to reverse. This persistence is often accompanied by ongoing airway injury. Nearly 20% of people with CF still experience pulmonary exacerbations requiring intravenous antibiotics annually, even while on CFTRm [101]. This suggests that correction of epithelial dysfunction alone is insufficient to reverse entrenched airway remodeling or eliminate matrix-protected bacterial populations [102].

These findings highlight a key distinction between prevention and reversal. CFTRm may reduce the likelihood of microbial establishment, but they can have limited impact when long-standing structural and ecologic changes are present [101,103]. Consequently, antimicrobial and adjunctive strategies remain necessary in individuals with established disease [104].

Importantly, a subset of pwCF, particularly those with class I and VII variants, remain ineligible for CFTRm, underscoring ongoing disparities in treatment response and access [105].

## 8. Therapeutic Strategies

Early eradication strategies have been shown to be effective when initiated promptly after pathogen detection, significantly reducing the risk of progression to chronic infection [61] (Table 1). This is supported by randomized controlled trial evidence demonstrating that early antibiotic eradication is achievable, whereas established chronic infection is rarely reversible [106]. Failure at this stage allows progression toward a more resilient disease state.

In established disease, treatment goals shift toward controlling microbial burden and limiting tissue damage rather than achieving complete eradication. Long-term inhaled antibiotics reduce bacterial density and exacerbation frequency but do not eliminate protected bacterial populations [107,108]. In chronic disease, bacteria inhabit structured communities and modified metabolic states that diminish antibiotic susceptibility. In this context, standard in vitro antibiotic susceptibility testing may not reliably predict clinical response, as treatment outcomes are influenced by the airway environment and bacterial adaptation [61].

Importantly, the reduced effectiveness of antibiotics in chronic infection does not reflect intrinsic inefficacy but rather the altered biologic context in which bacteria reside. Factors such as limited drug penetration, metabolic heterogeneity, and adaptive bacterial states contribute to incomplete bacterial clearance. These features are not fully captured by standard in vitro susceptibility testing, which may overestimate clinical efficacy. Furthermore, clinical response may occur despite in vitro resistance, underscoring the limitations of conventional resistance definitions in chronic CF infections [61]. Long-term inhaled antibiotic therapy is therefore widely used as a suppressive strategy. Agents such as tobramycin, colistin, and aztreonam have been shown in clinical studies to improve lung function, reduce exacerbation frequency, and enhance quality of life in pwCF with chronic *P. aeruginosa* infections. The use of inhaled antibiotics is now considered a standard of care in chronic infection, supported by substantial clinical trial evidence demonstrating improvements in pulmonary function tests and reductions in exacerbations [61].

A critical advancement in defining eradication protocols is the finding that intensive intravenous (IV) therapy does not offer superior clinical or microbiological outcomes over oral regimens for initial *P. aeruginosa* infection. The TORPEDO trial demonstrated that 14 days of IV ceftazidime and tobramycin was not superior to a 12-week course of oral ciprofloxacin in terms of sustained eradication at 15 months, provided that both groups received inhaled colistimethate sodium. Furthermore, oral treatment was found to be significantly more cost-effective, with a mean cost difference of nearly £6000 lower per patient [106,109]. Consequently, oral ciprofloxacin remains a cornerstone of early eradication; IV therapy should be reserved for severe exacerbations where clinical stability cannot be achieved through oral or inhaled routes [61,110]. Macrolides, particularly azithromycin, provide additional benefit through immunomodulatory and signaling effects rather than direct antimicrobial activity. Their use has been associated with reduced exacerbation rates and improved clinical stability [111].

Taken together, these findings indicate that antimicrobial therapy remains highly effective in early infection and clinically beneficial in chronic disease, although its role shifts from eradication to control. This paradigm aligns with current evidence-based CF management strategies, which emphasize adaptation of treatment according to infection stage, pathogen characteristics, and host airway environment [61]. This distinction underscores the need for stage-specific treatment strategies and supports the integration of antimicrobial therapy with approaches targeting microbial adaptation and host response [112].

In addition, the first years of life represent a critical ‘window of opportunity’ where microbial succession and infection history set the trajectory for lifelong lung function. Given that early-life dysbiosis can permanently alter pulmonary immune training, therapeutic strategies should increasingly focus on early intervention before irreversible structural remodeling and pathogen dominance occur [113].

**Table 1:** Summary of Therapeutic Strategies and Mechanisms Against *P. aeruginosa* in CF.

Strategy Phase and Category	Active Compound(s)	Mechanism of Action	MIC50/MIC90 (mg/L)	Key Reference(s)
I. Eradication Therapy (Early Infection)				
Inhaled Aminoglycoside	Tobramycin (TIS)	Inhibits protein synthesis by binding to the 30S ribosomal subunit.	1/16–2/32	[114,115,116,117]
Inhaled Polymyxin + Oral Quinolone	Colistin + Ciprofloxacin	Cell membrane disruption (Colistin) and DNA gyrase-topoisomerase IV inhibition (Cipro).	Colistin, 0.5/1–1/4; Cipro, 1/8–2/8	[114,115,116]
Intravenous (IV) Combination	Ceftazidime + Tobramycin	Inhibits cell wall synthesis (Ceftazidime)	2/64–4/64	[116,117]
Broad-Spectrum IV Beta-Lactams	Piperacillin-Tazobactam/Cefepime	Inhibits cell wall synthesis by binding to PBPs	Piperacillin-Tazobactam, 4/128–8/256; Cefepime, 4/8–>128	[116,117,118]
IV Carbapenem	Meropenem	High-affinity PBP binding; effective against many resistant strains.	0.25/16–1/32	[116,117,118]
Beta-lactam Combinations	Ceftazidime-Avibactam	Cephalosporin/BLI; Avibactam protects ceftazidime from Class A, C, and some D enzymes	2/4–2/8	[116,117,118,119]
Beta-lactam Combinations	Ceftolozane-Tazobactam	Novel Beta-lactam/BLI; Ceftolozane has high PBP affinity and AmpC stability	1/2–1/16	[114,116,117,120]
Beta-lactam	Cefiderocol	Siderophore cephalosporin; enters cell via iron transporters to inhibit PBPs	0.12/2–1/6	[114,119,121]
II. Suppressive Therapy (Established Chronic Infection)				
Maintenance Aminoglycoside	Tobramycin (TNS or DPI)	Sustained reduction in bacterial density and preservation of FEV1	1/16–2/32	[114,115,116,117,118,120,122]
Maintenance Polymyxin	Inhaled Colistin	Acts as a cationic detergent to disrupt the bacterial outer membrane.	0.5/1–1/4	[114,115,116]
Inhaled Monobactam	Aztreonam Lysine	Binds to PBP3 to inhibit cell wall synthesis; targets chronic populations.	8/64–8/128	[116,117]
III. Adjuvants and Emerging Biologics				
CFTR Modulators	ETI	Restores ion transport, improves MCC, and normalizes airway pH to reduce pathogen niches.		[123]
Anti-inflammatory/Immunomodulatory therapy	Long-term azithromycin	Immunomodulatory and anti-biofilm effects; quorum sensing interference		[124,125]
Mucolytic therapy/biofilm-disrupting adjunct	Dornase alfa	Disrupts biofilm eDNA; ↓ mucus viscosity; ↑ airway clearance		[126,127]
Iron-Mimetic Therapy	Gallium Nitrate	Mimics iron to disrupt bacterial metabolism and starve the pathogen.		[67]
Bacteriophage Therapy	AP-PA02/BX004-A Cocktails	Target-specific lysis of MDR strains; disrupts biofilm to improve drug access.		[44]
Persistence-Targeting Phage	Phage Paride	Hijacks (p)ppGpp circuits to replicate in and kill deep-dormant persister cells.		[44]
Biofilm Modulators	D-amino acids (D-Met, D-Trp)	Triggers biofilm disassembly by interfering with matrix stability.		[106,128]

Abbreviations: BLI, Beta-lactam inhibitor; CF, Cystic fibrosis; DPI, Dry powder inhaler; eDNA, Extracellular DNA; ETI, Elexacaftor/tezacaftor/ivacaftor; FEV1, Forced expiratory volume in 1 s; IV, Intravenous; MCC, Mucociliary clearance; MDR, Multidrug-resistant; MIC, Minimum inhibitory concentration; PBP3, Penicillin-binding protein 3; TIS, Tobramycin inhalation solution.

### 8.1. Vaccine Development Strategies

Development of an effective vaccine against *P. aeruginosa* remains challenging due to antigenic variability and adaptive capacity. Early approaches targeting single antigens, such as O-antigen polysaccharides, flagellar components, or outer membrane proteins, have shown limited success. Although some of these candidates generated immune responses in clinical trials, they failed to demonstrate consistent protection against infection. These findings suggest that targeting a single virulence determinant may be insufficient for preventing infection by a highly adaptable pathogen [129,130].

Recent advances in genomic technologies have enabled alternative approaches, particularly reverse vaccinology. This strategy uses genome wide analyses to identify conserved surface exposed or secreted antigens that could serve as vaccine targets. Using this approach, multiple conserved candidate proteins have been identified across diverse *P. aeruginosa* strains, including those isolated longitudinally from individuals with CF [131]. Experimental studies in murine models demonstrated that combinations of selected antigens could significantly increase survival and reduce bacterial burden following pulmonary infection. These findings suggest that multivalent vaccine strategies might offer greater protective potential than single antigen formulations [131].

Another promising strategy involves targeting virulence mechanisms rather than bacterial viability. Vaccines directed against components of the T3SS, quorum sensing regulators, or biofilm associated structures aim to attenuate bacterial pathogenicity and enhance host immune clearance [132,133]. Such antivirulence vaccination strategies could theoretically reduce selective pressure for antibiotic resistance while limiting tissue damage during infection.

Innovative inhaled delivery systems are being developed to overcome the physical barriers of the CF lung. Particle engineering for dry powder inhalers uses functional additives such as leucine to improve aerodynamics and reduce moisture sensitivity. Furthermore, the incorporation of D-amino acids (e.g., D-methionine, D-tryptophan) has shown potential in enhancing antibiofilm activity when combined with ciprofloxacin, offering a strategy to improve drug penetration into recalcitrant aggregates [128].

In the context of CF, vaccine development presents additional challenges. Chronic colonization often occurs early in life, and adaptive bacterial evolution within the CF airway results in phenotypically diverse populations. Therefore, effective vaccines may need to be administered prior to initial colonization or designed to target conserved mechanisms essential for bacterial persistence. Continued advances in systems biology, immunology, and structural vaccinology may ultimately facilitate the development of next-generation vaccines capable of preventing or delaying *P. aeruginosa* colonization in susceptible populations.

### 8.2. Anti-Inflammatory Therapeutic Strategies

Antimicrobial therapies target microbial burden; however, host-mediated tissue injury remains a major determinant of disease progression in CF. Therapeutic approaches therefore increasingly aim to modulate dysregulated immune responses. More targeted anti-inflammatory strategies are currently under investigation. Inhibition of NE has gained attention due to its strong association with lung function decline [134]. Similarly, modulation of inflammasome pathways, particularly NLRP3 activation [135], has been proposed as a potential therapeutic target due to its role in promoting IL-1β-mediated inflammation in CF airways [22].

Specialized pro-resolving lipid mediators, including resolvins and lipoxins, have been shown to actively terminate inflammatory responses and promote tissue repair [136]. Targeting upstream activation of neutrophils represents another promising therapeutic direction. In this context, DPP-1 inhibitors such as brensocatib reduce neutrophil serine protease activity and may attenuate tissue damage in chronic respiratory diseases [137].

Importantly, anti-inflammatory therapies must be carefully balanced with host defense mechanisms. Excessive immunosuppression could impair bacterial clearance and exacerbate infection. Therefore, future therapeutic strategies will likely focus on restoring immune homeostasis rather than broadly suppressing inflammation. Integrating targeted anti-inflammatory interventions with antimicrobial therapy and CFTRm may ultimately provide the most effective strategy for mitigating progressive lung damage in CF.

### 8.3. Bacteriophage Therapy

Bacteriophage therapy has recently re-emerged as a promising alternative or adjunct strategy for the treatment of chronic bacterial infections, particularly in the context of increasing antibiotic resistance. Bacteriophages are viruses that specifically infect and lyse bacterial cells, offering a highly targeted antimicrobial approach that differs fundamentally from conventional antibiotics. In CF, where *P. aeruginosa* frequently develops multidrug resistance and persists within biofilms, phage therapy has attracted significant attention as a potential therapeutic tool [138].

Recent clinical investigations provided encouraging preliminary evidence for the feasibility of phage therapy in CF. In compassionate use studies, personalized nebulized bacteriophage therapy targeting patient-specific *P. aeruginosa* strains has resulted in reductions in bacterial density within sputum samples and modest short-term improvements in lung function without significant disruption of the overall airway microbiome. These findings suggest that phage therapy may offer a targeted approach for controlling persistent infections, particularly in patients with multidrug-resistant organisms [139,140,141].

However, several biologic and clinical challenges remain. *P. aeruginosa* populations in the CF airway are genetically heterogeneous, which may require personalized phage cocktails to ensure effective bacterial targeting. Additionally, temperate prophages naturally integrated within bacterial genomes can influence bacterial susceptibility to therapeutic lytic phages and may alter antibiotic sensitivity during chronic infection. These complex host phage bacteria interactions highlight the need for individualized treatment strategies and further mechanistic research [141,142].

While lytic phages generally require metabolically active hosts, the discovery of phages such as phage Paride offers a path to targeting deep-dormant, antibiotic-tolerant cells. Paride hijacks dormancy-associated regulatory circuits, such as the (p)ppGpp stringent response and the sigma factor RpoS, allowing replication within quiescent bacteria. It also releases signals that “awaken” neighboring cells, rendering them susceptible to beta-lactam antibiotics. However, humoral immunity remains a hurdle; neutralizing anti-phage antibodies (IgG, IgA, IgM) can emerge within 6–35 days of therapy, potentially reducing bioavailability and necessitating “stealth” engineering strategies such as PEGylation or membrane cloaking to evade host detection [44].

Future advances in phage engineering, synthetic biology, and personalized medicine may further enhance the therapeutic potential of bacteriophages. Engineered phages capable of delivering antimicrobial payloads, disrupting biofilm matrices, or targeting specific bacterial virulence mechanisms are currently under investigation. Although large scale clinical trials are still limited, bacteriophage therapy represents a promising component of next-generation antimicrobial strategies aimed at controlling chronic *P. aeruginosa* infection in CF.

### 8.4. Limitations of Current Therapeutic Strategies

Despite major advances in CF management, several limitations remain. CFTRm improves epithelial function but does not reliably eliminate established infection. Their benefit is therefore greater in prevention than in reversal of advanced disease [143]. A major diagnostic and therapeutic challenge in chronic infection is the presence of heteroresistance. Clinical isolates often contain unstable subpopulations of cells with increased antibiotic resistance that standard susceptibility tests fail to detect. This phenotype, often driven by the transient overexpression of efflux pumps or loss of OprD porins, can lead to unexplained treatment failure and the rapid regrowth of resistant populations once antibiotics are withdrawn [67].

Clinical evidence for emerging therapies is still limited. Many approaches are supported by small or early-phase studies, and robust long-term data are lacking. Experimental models also remain insufficient. In vitro systems and animal models do not fully capture the complexity of the human airway, limiting translation of findings into clinical practice [144,145,146].

Finally, the polymicrobial nature of the CF airway complicates treatment. Interactions between microbial communities and the host may influence therapeutic response in ways that are not yet fully understood.

## 9. Conclusions

This review highlights that CF lung disease is best understood as a dynamic process of host–pathogen co-adaptation. In this context, epithelial dysfunction, immune dysregulation, and microbial persistence are tightly interconnected. By integrating current evidence across molecular, immunological, and clinical domains, this review provides a comprehensive framework for understanding disease progression and identifying potential therapeutic targets.

Despite advances in CFTRm, these therapies do not fully reverse airway microenvironment alterations or eliminate established *P. aeruginosa* persistence, and current antimicrobial strategies remain largely suppressive rather than curative.

Future progress hinges on early intervention prior to the establishment of chronic infection and integrated approaches that target epithelial dysfunction, microbial persistence, and immune dysregulation. Alignment of translational research, clinical trials, and patient-centered care will be critical to efficiently translate these strategies into improved clinical outcomes.

## Data Availability

No new data were created or analyzed in this study.

## Abbreviations

The following abbreviations are used in this manuscript:

ASL	airway surface liquid
CF	cystic fibrosis
CFTR	cystic fibrosis transmembrane conductance regulator
CFTRm	cystic fibrosis transmembrane conductance regulator modulator therapy
Cl^−^	chloride
DAMPs	damage-associated molecular patterns
DPP-1	dipeptidyl peptidase-1
ENaC	epithelial sodium channel
ETI	Elexacaftor/tezacaftor/ivacaftor
HCO_3_^−^	bicarbonate
MCC	mucociliary clearance
Na^+^	sodium
NETs	neutrophil extracellular traps
NHERF1	Na^+^/H^+^ exchanger regulatory factor-1
pwCF	people with CF
*P. aeruginosa*	*Pseudomonas aeruginosa*
PAMP	pathogen-associated molecular patterns
ROS	reactive oxygen species
*S. aureus*	*Staphylococcus aureus*
Treg	regulatory T cells
